# Case of Ante Version of the Uterus during Parturition

**Published:** 1850-12

**Authors:** William Treat

**Affiliations:** Buffalo


### ART. II.—*Case of Ante version of the Uterus during Parturition.* By William Treat, M. D.

Having met with a case of this accident recently, and not calling to mind any previous reading of the liability of its occurrence, after taxing mother-wit to a successful issue, I was tempted to refer to such authority in

Obstetrics as I had near at hand, and Blundell is of the number, without finding mention made of anteversion under any circumstances, save in James Burns, wherein a brief paragraph appears. After speaking of retroversion, he adds:

"The Uterus is also sometimes anteverted, that is, the fundus is thrown forward so as to compress the neck of the bladder, and its mouth is turned to the sacrum. Of this accident I have never seen an instance during gestation, and from the nature of the case it must be very rare; but I have met with it, from enlargement of the fundus uteri, in the unimpregnated state." He adds, in a note, that, "Baudelocque relates a case from Chopart where it, (anteversion,) was produced in the second month of pregnancy by the action of an emetic."

The curious reader may find other cases elsewhere related, undoubtedly, but I have not leisure or inclination for the present to seek further. Believing the case I have recently had in charge possesses sufficient interest, on suggestion from others, I am induced to write a brief description for publication.

*October* 25–6*th*—At night I was called, as by previous arrangement, to see Mrs. H——, aged 28, a thick-set, short-waisted woman, about 5 feet in height, in labor with her fourth child. When making the first vaginal examination, although the pains were not very active, the progress of the labor was easily determined. After an hour's delay, in the act of making a second examination, an unusual quantity of amniotic fluid was discharged before the finger had reached the membranes. Immediately the presentation of the child was ascertained to be that of the head, with the face towards the sacrum, and with it, the os uteri was found to be dilated two or more inches in diameter. As the pains were not long protracted or of frequent occurrence, I left the bed side and reclined upon a lounge, not for sleep, but to "but to bide my time," and, about a half an hour after, again repeated the examination,—the woman upon the left side, in bed,—when to my surprise although the woman was suffering from much parturient pain the uterus could scarcely be reached, and, when circumscribed by the finger, the mouth had disappeared as though occluded. Placing my left hand upon the abdomen for the purpose of pressing its walls in such way as to bring the uterus down and nearer to the reach of the index finger of the right hand, the difficulty was immediately ascertained; *the fundus of the uterus was deflected over the pubes at least half the length of the gravid uterus!* and the child thus thrown upon its back was at each contracting effort of the uterus not only "making haste slowly," but with an uphill and retrograde movement!

The patient was now placed on her back, and an effort immediately made to replace the uterus by seizing the fundus during the intermission of pain, and sliding it under the abdominal parieties, upwards and forwards to a place where it was at right angles with the body of the patient. At this point the resistance, it was thought, could not safely be overcome by reason of the super-imposed viscera, resisted by their proximity to the diaphragm, the head and shoulders of the woman having been previously depressed to overcome gravity. Now it was that the anterior edge of the dilated os uteri, at each uterine contraction, could be felt, as also a portion of the vertex of the presenting head. Yet with each contractile act was the uterus, in greater or less degree, renewedly anteverted, although effort was made sometimes with one hand, oftener with both, upon the abdomen to suppress it. After some delay, finding the pains becoming more violent and frequent, and the labor making little or no progress, the bandage usually designed for after purpose was applied by passing one end under the back, and there fastened, while the other was brought round and forward over the abdomen and held by an assistant in such a manner as to make firm and continuous pressure upon the fundus uteri in a line upwards and backwards. Thereafter, with but little more difficulty, the head and body of the child performed a gradual evolution and advanced, as in natural labor, and thus relieved me, after only six hours detention, from what for a time indicated a necessity for instrumental aid. With this happy result terminated my chief interest in this, if not novel, certainly precocious exhibition of "a distinguished stranger" in the art of "grand and lofty tumbling." The nurse soon announced the weight at 9½ lbs.—¼lb. less than its immediate predecessor.*

The cause of this unlooked for and unexpected position of the uterus, during labor, is doubtless attributable to the short body and by consequence great rotundity of the female,—bearing as she did, a large child with an excess of amniotic fluid,—the sudden discharge of the latter depriving the uterus of the requisite support from the abdominal muscles, now not in full tone, all of which combined to facilitate the over-turning of the matrix and its contents.

Buffalo, Nov. 6th, 1850.

---

*While upon the subject of midwifery, having here incidentally mentioned the weight of the child, I am tempted to believe that by way of "big babies," as in every thing else, a Yankee's word for it, in "the States" the women "beat all creation." I am led to this remark by long observation, after having read and kept in mind the statistical tables of weight made with the "greatest accuracy" at the Matron's Hospital, Paris, wherein of 7,077 cases, it is stated, only three weighed 10 pounds, eighty-two from 9 to 9½ lbs., 463 from 8 to 8¾ lbs., and about one-half weighed only from 6 to 6¾lbs.

